# S15. ABNORMAL EYE TRACKING IN PATIENTS WITH SCHIZOPHRENIA UNDER THE SOCIAL SCENE

**DOI:** 10.1093/schbul/sby018.802

**Published:** 2018-04-01

**Authors:** Qijing Bo, Xianbin Li, Wenlong Jiang, Changming Wang, Chuanyue Wang

**Affiliations:** Beijing Anding Hospital, Capital Medical University

## Abstract

**Background:**

To investigate whether the eye movement pattern is different between facial emotion recognition and real social scene emotion recognition, and which can better reflect the social function and the clinical symptoms using a novel theme identification task.

**Methods:**

Total 29 patients with schizophrenia and 31 healthy controls completed the theme identification task, in which subjects selected which word, out of positive, neutral and negative, described the theme of a picture under facial emotion recognition and real social scene emotion recognition. Positive and negative syndrome scale (PANSS) and social function in psychosis inpatients (SSPI) were used to assess the symptom and social function.

**Results:**

The schizophrenia’s eye movement paradigms under both facial emotion and social scene show decreased number of fixation (t=-3.49, P=0.00; t=-3.62, P=0.00), decreased number of saccades (t=-3.15, P=0.00; t=-3.72, P=0.00), decreased scan path length (t=-2.23, P=0.03; t=-4.18, P=0.03), decreased fixation number in interest area (t=3.01, P=0.00; t=-3.24, P=0.00). Different from facial emotion cognition, the eye movement under social scene cognition showed lower percentage of fixation number in interest area than that in healthy subjects (P=0.01), furthermore, the length of scan path under the negative social scene pictures was associated with the total score of SSPI (r=-0.38, P=0.04), the PANSS total score (r=-0.46, P=0.01), the positive symptoms score (r=-0.39, P=0.04), the general score (r=-0.50, P=0.01).

**Discussion:**

The patients showed more abnormal eye tracking indicators under social scene than facial emotion. Under negative emotion social scene, the length of scan path related to social function and clinical symptoms, it may be a potential indicator to evaluate social function and degree of disease.

